# Distinct transcriptional profiles of ozone stress in soybean (*Glycine max*) flowers and pods

**DOI:** 10.1186/s12870-014-0335-y

**Published:** 2014-11-28

**Authors:** Courtney P Leisner, Ray Ming, Elizabeth A Ainsworth

**Affiliations:** Department of Plant Biology, University of Illinois, Urbana-Champaign, Urbana, IL 61801 USA; USDA ARS Global Change and Photosynthesis Research Unit, 1201 W. Gregory Drive, Urbana, IL 61801 USA

**Keywords:** Oxidative stress, *Glycine max*, RNA-Sequencing, Matrix metalloproteinases, Cell wall modification

## Abstract

**Background:**

Tropospheric ozone (O_3_) is a secondary air pollutant and anthropogenic greenhouse gas. Concentrations of tropospheric O_3_ ([O_3_] have more than doubled since the Industrial Revolution, and are high enough to damage plant productivity. Soybean (*Glycine max* L. Merr.) is the world’s most important legume crop and is sensitive to O_3_. Current ground-level [O_3_] are estimated to reduce global soybean yields by 6% to 16%. In order to understand transcriptional mechanisms of yield loss in soybean, we examined the transcriptome of soybean flower and pod tissues exposed to elevated [O_3_] using RNA-Sequencing.

**Results:**

Elevated [O_3_] elicited a strong transcriptional response in flower and pod tissues, with increased expression of genes involved in signaling in both tissues. Flower tissues also responded to elevated [O_3_] by increasing expression of genes encoding matrix metalloproteinases (MMPs). MMPs are zinc- and calcium-dependent endopeptidases that have roles in programmed cell death, senescence and stress response in plants. Pod tissues responded to elevated [O_3_] by increasing expression of xyloglucan endotransglucosylase/hydrolase genes, which may be involved with increased pod dehiscence in elevated [O_3_].

**Conclusions:**

This study established that gene expression in reproductive tissues of soybean are impacted by elevated [O_3_], and flowers and pods have distinct transcriptomic responses to elevated [O_3_].

**Electronic supplementary material:**

The online version of this article (doi:10.1186/s12870-014-0335-y) contains supplementary material, which is available to authorized users.

## Background

Current tropospheric O_3_ concentrations ([O_3_]) are estimated to cost $14 to $26 billion in annual global crop economic losses [1] and severely impact human health, accounting for an estimated 0.7 million deaths per year [2]. Ozone in the troposphere is formed through the photochemical oxidation of volatile organic compounds (VOCs), carbon monoxide and methane in the presence of nitrogen oxides (NO_x_) [3]. Ozone is a dynamic pollutant and concentrations vary temporally and spatially, with higher concentrations in the Northern Hemisphere compared to the Southern Hemisphere, and typically higher [O_3_] in the summer compared to the winter [3]. Background tropospheric [O_3_] have more than doubled since the Industrial Revolution and are projected to increase by an additional ~20% by the year 2100 if current high emission rates continue [4]. In the crop growing regions of the Northern Hemisphere, summer concentrations of O_3_ often exceed 40 ppb, which exceeds the critical threshold for damage to sensitive crops, including soybean (*Glycine max*) [5].

When taken up by plants, O_3_ is converted into other reactive oxygen species (ROS), and can induce signaling pathways that lead to programmed cell death, especially with exposure to very high [O_3_] [6]. At lower concentrations, chronic exposure to elevated [O_3_] decreases photosynthetic carbon assimilation and stomatal conductance, and accelerates the process of senescence [7,8]. In addition to leaf-level effects, O_3_ negatively impacts plant fitness and reproductive development, which can be mediated through reduced carbon allocation from source tissues and/or through direct effects on reproductive tissues [9,10]. A meta-analysis of published studies from 1968 to 2010 of O_3_ effects on plant reproductive processes reported that exposure to elevated [O_3_] decreased seed number and seed size, as well as fruit number and fruit size when compared to plants grown in charcoal-filtered, O_3_-free air [11]. However, the meta-analysis also showed that elevated [O_3_] did not significantly alter inflorescence number, flower weight or flower number [11]. This suggests that plants can compensate to some extent from O_3_ damage [12], and also that the effects of O_3_ can be tissue-specific.

**Figure 1:**
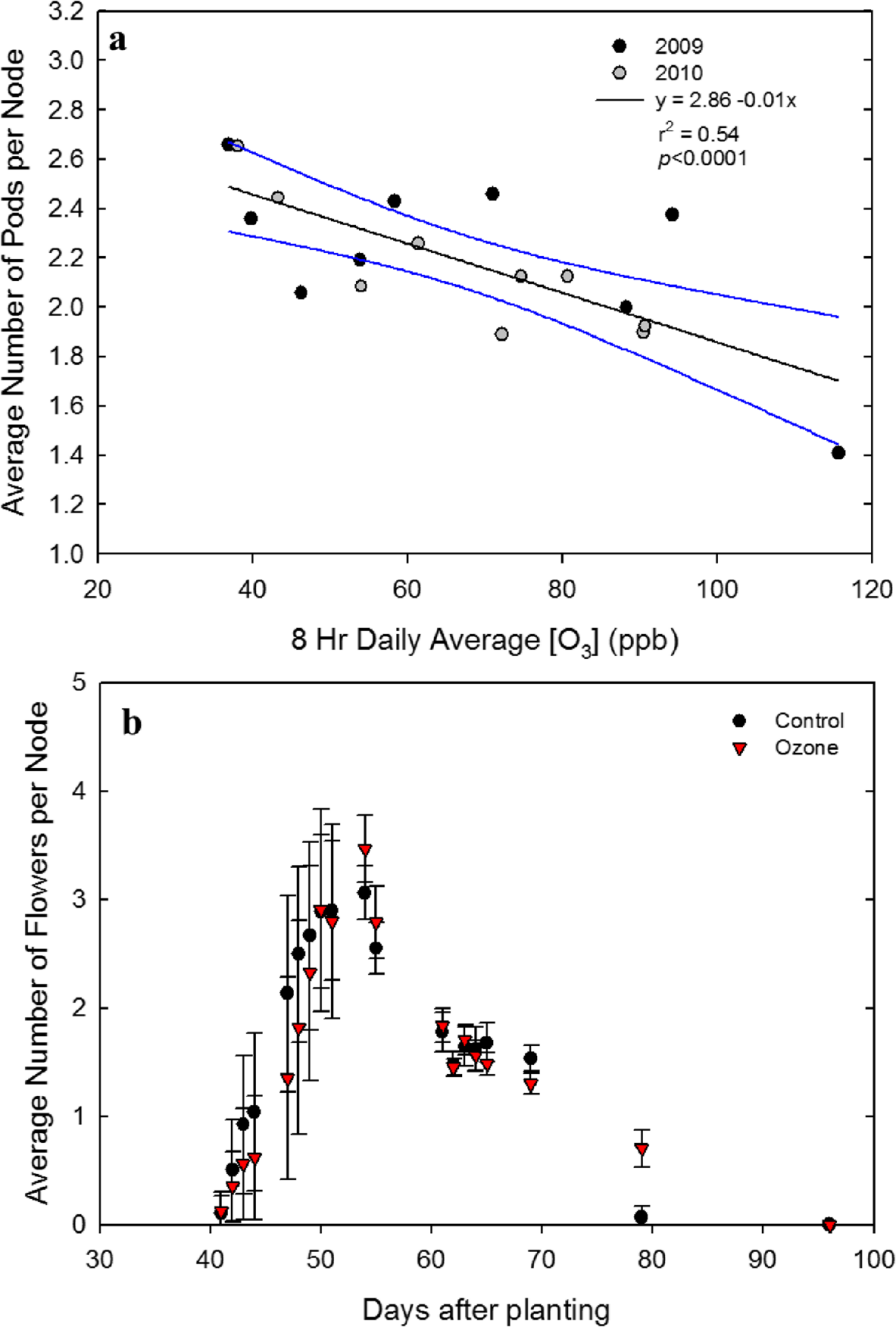
**The effect of O**_**3**_ **on the number of flowers and pods produced per node in field-grown soybean. (a)** Linear regression of the average number of pods per node for soybean plants grown under eight [O_3_] at the SoyFACE facility (http://www.igb.illinois.edu/soyface/) in Champaign, Illinois in 2009 and 2010. Blue lines show the 95% confidence intervals. Experimental design, planting conditions, meteorological data and harvesting methods are found in [27]. **(b)** Average flower number per node for soybean plants grown under ambient (44 ppb) and elevated (100 ppb) [O_3_] at the SoyFACE facility in 2011. Flower number per node was monitored daily for five plants per ambient and elevated [O_3_] plot (n = 2 for ambient, n = 4 for elevated [O_3_]).

Soybeans have naturally high levels of floral and pod loss, and subsequent seed and yield loss is greatest when stress occurs during flower and early pod development [13]. Flower and pod abscission can range from 32 to 82% in soybean [14-16], but this varies considerably with location on the plant [15-17], location in the canopy [18], source-sink relations [19], hormone levels [13,20,21], shade [22] and water status [13,23,24]. Ethylene promotes flower and pod abscission in soybean [25], and elevated [O_3_] can increase ethylene emission in plants [26]. Therefore, elevated [O_3_] has the potential to increase flower and pod abscission. In field-grown soybean exposed to elevated [O_3_] for an entire growing season [27], pod production was decreased by elevated [O_3_], but flower number was not affected (Figure 1). Based on this evidence from the field, it is hypothesized that the transcriptional responses of soybean flowers and pods to elevated [O_3_] would be distinct.

Previous studies have examined changes in transcript abundance in plants in response to elevated [O_3_] [6,28-35]; however, most of these studies have focused on leaves. In soybean, both flower and pod tissues also have stomata through which O_3_ could enter and elicit a signaling response [36,37]. Next-generation sequencing technology allows examination of changes to the entire transcriptome, which could facilitate interpretation of the complex phenotypes that underpin O_3_ response in plants. By investigating how elevated [O_3_] affects the transcriptome of reproductive tissues, we can begin to understand the distinct responses in different tissues and identify potential targets for improving tolerance. Therefore, in this study, the transcriptome of flower and pod tissue from chamber-grown soybean plants at ambient (<20 ppb) and elevated [O_3_] (150 ppb) was investigated. Both flower and pod tissues showed significant transcriptomic responses to elevated [O_3_]. While 277 transcripts were responsive to elevated [O_3_] in both tissues, most of those transcripts did not change in the same direction or at the same magnitude in flowers and pods, indicating that the transcriptional response to O_3_ in different reproductive tissues was distinct.

## Results and discussion

### Overlapping effects of elevated [O_3_] on the transcriptome of flower and pod tissue in soybean

**Figure 2:**
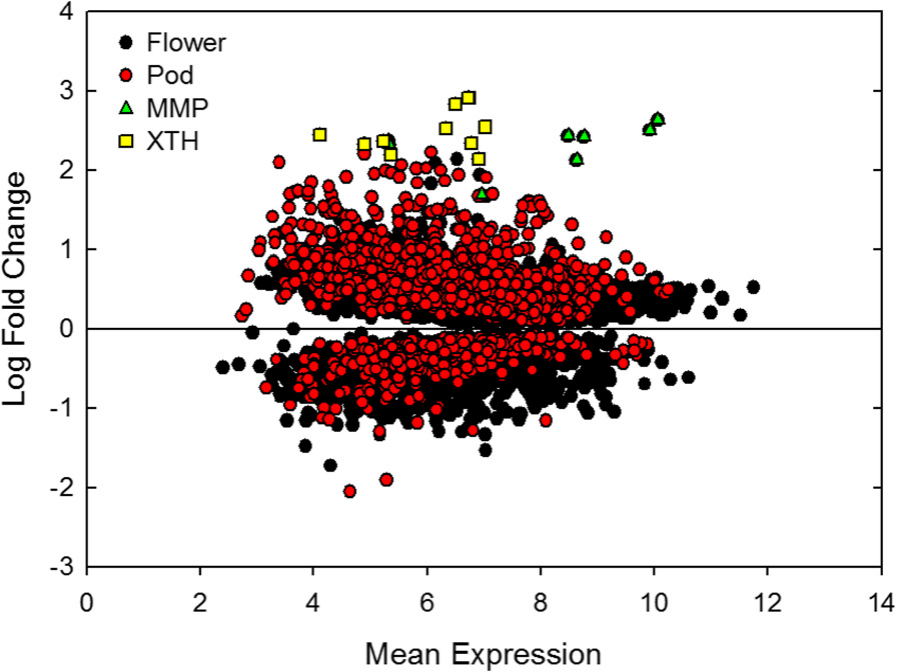
**Comparison of differential gene expression in flower and pod tissue under elevated [O**_**3**_ **].** The log fold change for all genes differentially expressed in flower and pod tissue (*p* <0.05) was plotted against the mean expression value for that gene measured in both ambient and elevated [O_3_]. Black circles represent genes differentially expressed in flowers and red circles represent genes differentially expressed in pods. Green triangles represent MMP genes differentially expressed in flowers. Yellow squares represent XTH genes differentially expressed in pods. Reference line represents a log fold change of zero. Values above the reference line are genes increased in abundance compared to ambient [O_3_] and values below the reference line are genes decreased in abundance compared to ambient [O_3_].

**Figure 3:**
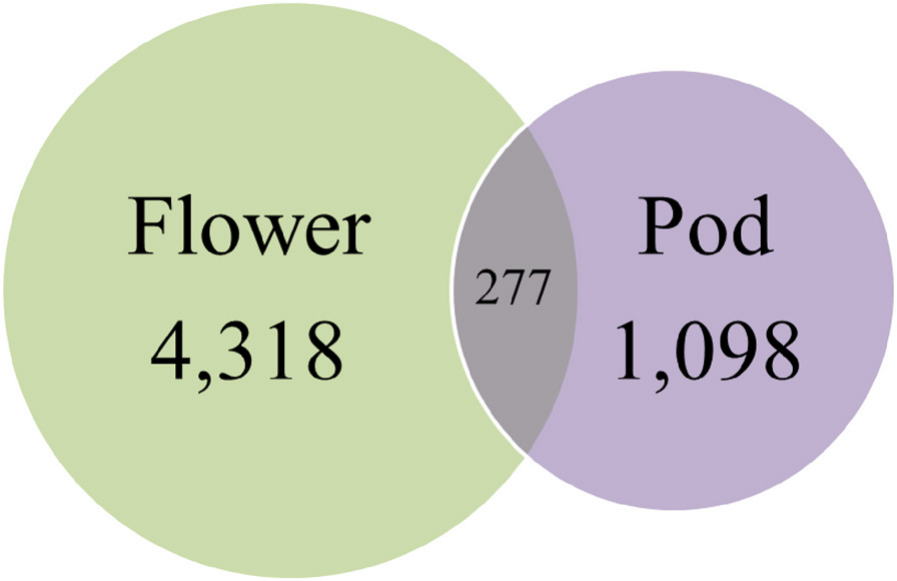
**Venn diagram of differentially expressed genes in flower and pod tissues in response to elevated [O**_**3**_ **].** Numbers of genes that were differentially expressed in response to elevated [O_3_] in flowers (green), pods (purple) and in both tissues (overlapping).

Flower and pod development in soybean are sensitive to environmental stress [23,24,38,39], and elevated [O_3_] significantly impacted pod production, but not flower production (Figure 1). In order to identify the genetic mechanisms underpinning O_3_ response in soybean pods and flowers, the transcriptome of flower and pod tissues was compared using RNA-Sequencing (RNA-Seq). The global transcriptional analysis showed the magnitude of potential responses to elevated [O_3_] in flowers and pods was similar, with genes showing approximately the same range of both mean expression values in flowers and pods, and similar potential log fold change responses to elevated [O_3_] in the two tissues (Figure 2). However, more than three times as many genes were differentially expressed in flower tissue (4,595 genes) than in pod tissue (1,375 genes; Figure 3) in response to elevated [O_3_], and only 277 of those genes were differentially expressed in both flowers and pods (Figure 3).

**Figure 4:**
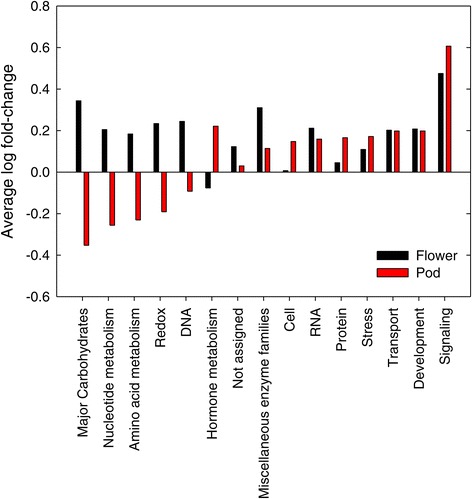
**Average fold change of genes differentially expressed in both flowers and pods in response to elevated [O**_**3**_ **].** Average log fold change of all genes within a functional category that significantly responded to elevated [O_3_] in both flowers (black bars) and pods (red bars). A positive log fold change indicates increased abundance in elevated [O_3_] compared to ambient [O_3_], while a negative log fold change indicates decreased abundance in elevated [O_3_].

**Figure 5:**
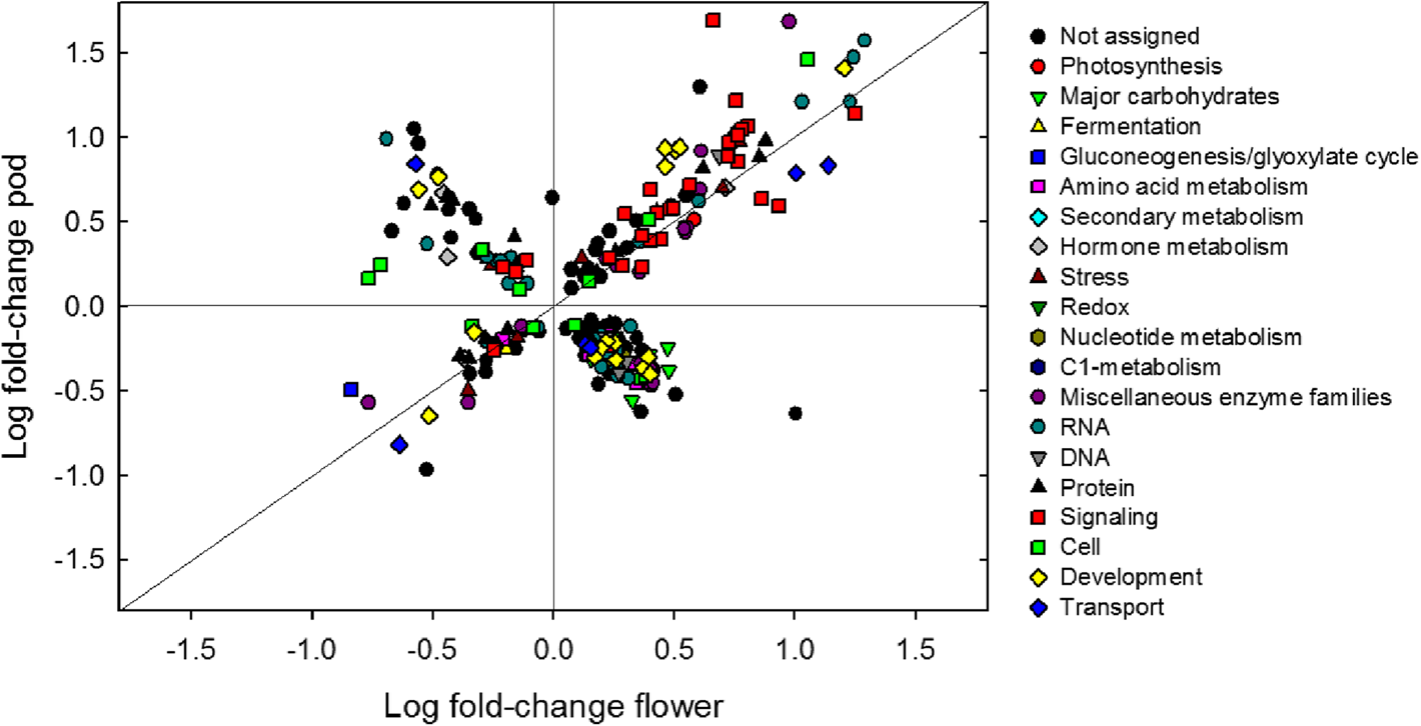
**Comparison of expression changes in response to elevated [O**_**3**_ **] in soybean flowers and pods.** The log fold change of the 277 individual genes significantly changing in response to elevated [O_3_] in both pods vs. flowers is shown. Functional groups are represented by different symbols/colors. The 1:1 line represents genes that have the same direction of fold change in flower and pod tissue.

Differentially expressed genes in pods and flowers were grouped into functional categories (Figure 4). Nine of 15 total functional categories showed pod and flower genes changing in the same direction in response to elevated [O_3_] (Figure 4). Transcripts involved in signaling, development, transport, stress, protein and RNA were expressed at greater levels on average in both pods and flowers exposed to elevated [O_3_] compared to control (Figure 4). While average changes in expression based on functional categories suggests that there was overlap in the transcriptional response of flowers and pods to elevated [O_3_], investigation of individual genes showed that there was not good correspondence of the direction or magnitude of the response (Figure 5). Less than half of the 277 genes that were significantly affected by elevated [O_3_] in both flowers and pods responded in a similar direction, with 78 of the 277 genes increasing in both tissues in response to elevated [O_3_] and 33 decreasing in both tissues in response to elevated [O_3_] (Figure 5). Many of the transcripts that fell on the 1:1 line in Figure 5 were involved in signaling and RNA processing, including 12 leucine-rich repeat receptor-like kinases (RLKs) and 3 cysteine-rich Domain of Unknown Function 26 (DUF26) RLKs (also known as cysteine-rich receptor-like kinases, CRK). Plant RLKs are transmembrane proteins involved in signal perception and form a large multi-gene family with regulatory roles in development, abiotic and biotic stress responses in plants [40,41]. Recent analysis of the response of Arabidopsis DUF26 RLKs showed that many of the 44 RLKs were specifically up-regulated in response to O_3_ stress in leaves [42], including DUF26 30 (CRK 26), DUF26 29 (CRK 29) and DUF26 41 (CRK 2), which also had a significant increase in expression in soybean pods and flowers exposed to elevated [O_3_]. Wraczek et al. [42] found that the general pattern of DUF26 expression responses to O_3_ was most similar to the transcriptional response to pathogen infection, which like O_3_ elicits an ROS burst in the apolost. The transcriptional response to O_3_ however, was very different from expression responses to high light treatments or chemical treatments that increased ROS production in chloroplasts or mitochondria [42]. Thus, it was further suggested that the DUF26 domain, which has a conserved cysteine motif C-8X-C-2X-C, could act as an apolastic ROS sensor [42].

A number of WRKY domain transcription factors also showed significantly greater expression under elevated [O_3_] in both soybean flowers and pods (Glyma04g40130, Glyma06g14720, Glyma14g36430, Glyma14g36438, Glyma14g36446). The WRKY transcription factor family is one of the largest families of transcription factors in plants, with 133 members in the soybean genome [43]. WRKYs function in many plant processes including response to biotic and abiotic stresses, and senescence [44]. Up-regulation of WRKY transcription factors in response to O_3_ stress has been previous reported, primarily in the leaves of trees [45-47]. Two of the WRKY transcription factors with increased expression in both flower and pod tissues in response to elevated [O_3_] (Glyma04g40130 and Glyma06g14720) were likely formed through a segmental duplication event that is estimated to have occurred 20 million years ago [43].

### Distinct effects of elevated [O_3_] on the transcriptome of flower and pod tissue in soybean

**Figure 6:**
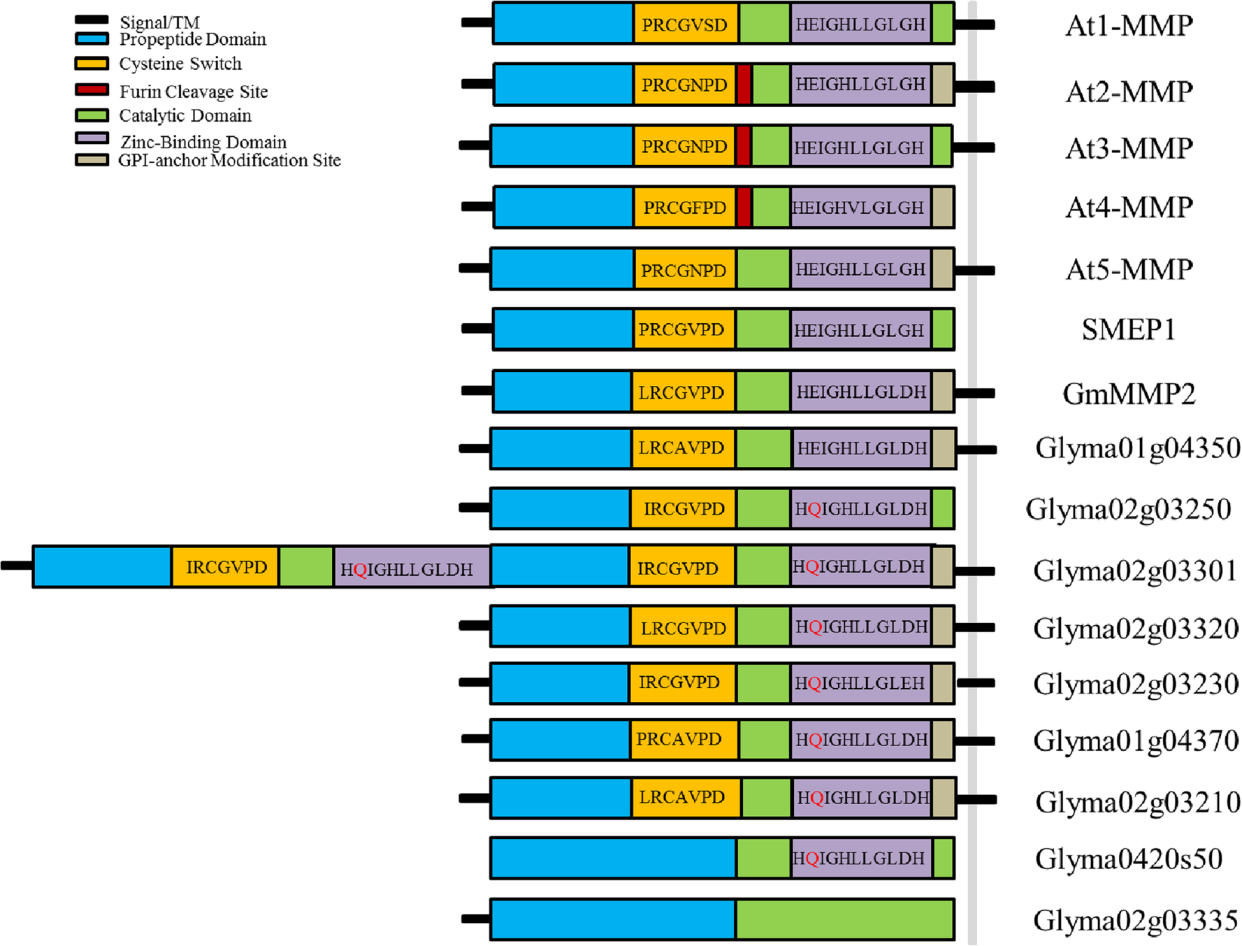
**Domain analysis of plant matrix metalloproteinase (MMP) genes.** General structure of known plant MMPs and putative MMPs identified in soybean flowers. The cysteine switch and zinc binding domain sequence motifs are shown for all genes (when present). The E to Q residue substitution in the zinc-binding motif of the catalytic domain is indicated in red.

Although there was some overlap in transcriptional responses to elevated [O_3_] in flowers and pods, the vast majority of genes changing in either tissue were distinct (Figure 3), and even among the genes that were expressed in both flower and pod tissues, the fold changes in expression were of different magnitudes or in the opposite direction (Figure 5). In flower tissue, the genes with the greatest increase in abundance in response to elevated [O_3_] included matrix metalloproteinase (MMP) genes and genes related to hormone metabolism and signaling (Table 1). Genes annotated as MMPs also had high mean expression levels (Figure 2). While little is known about their role in soybean flowers, in other tissues MMPs function in degradation of the extracellular matrix (ECM) in response to senescence, stress and programmed cell death [48-51]. Domain analysis indicated that 7 of the 9 differentially expressed genes annotated as MMPs had both a cysteine switch domain and a zinc-binding domain, both of which are required for characterization as a MMP (Figure 6) [52-54]. Those genes with both required domains were termed putative soybean flower MMP genes.

**Table 1:** **Genes with the greatest log fold change in response to elevated [O**_**3**_ **] in flower tissues**

**Gene**	***p*** **- value**	**Log fold change**	**Functional group**	**Description**
Glyma02g03250	0.018	2.63	Protein	Matrixin family protein
Glyma02g03301	0.019	2.51	Protein	Matrixin family protein
Glyma02g03320	0.023	2.43	Protein	Matrixin family protein
Glyma02g03230	0.021	2.42	Protein	Matrixin family protein
Glyma01g04370	0.024	2.37	Protein	Matrixin family protein
Glyma0420s50	0.024	2.32	Protein	Matrix metalloproteinase
Glyma02g03335	0.028	2.32	Protein	Matrix metalloproteinase
Glyma02g03280	0.026	2.14	NA	NA
Glyma02g03210	0.019	2.12	Protein	Matrix metalloproteinase
Glyma16g01990	0.019	2.09	Hormone metabolism	2-oxoglutarate (2OG) and Fe(II)-dependent oxygenase superfamily protein
Glyma07g32650	0.042	1.93	Protein	Cysteine proteinases superfamily protein
Glyma12g02410	0.024	1.83	Miscellaneous	Glycosyl hydrolase superfamily protein
Glyma01g04350	0.029	1.69	Protein	Matrix metalloproteinase
Glyma07g05420	0.017	1.67	Hormone metabolism	2-oxoglutarate (2OG) and Fe(II)-dependent oxygenase superfamily protein
Glyma08g21321	0.024	1.47	Signaling	Leucine-rich repeat receptor-like protein kinase
Glyma13g41330	0.015	1.46	Transport	ZIP Zinc transporter
Glyma15g04090	0.014	1.40	Transport	ZIP Zinc transporter
Glyma12g31250	0.028	1.37	NA	NA
Glyma16g28510	0.046	1.37	Signaling	Leucine-rich repeat receptor-like protein kinase
Glyma07g28940	0.038	1.33	Cell wall	BURP domain-containing protein
Glyma16g28530	0.030	1.32	Signaling	Leucine-rich repeat receptor-like protein kinase
Glyma14g37946	0.023	1.29	Cell	Exocyst subunit exo70 family protein B1
Glyma02g09181	0.041	1.27	Signaling	Leucine-rich repeat receptor-like protein kinase
Glyma16g28460	0.044	1.26	Signaling	Leucine-rich repeat receptor-like protein kinase
Glyma12g31280	0.025	1.21	NA	NA

‘NA’ indicated genes not assigned an annotation. FDR-adjusted *p*-values are shown.

The putative MMP gene Glyma02g03301 had two identical cysteine switch domains and zinc-binding domains, which was unique compared to the other putative flower MMP genes. All putative flower MMP genes had a signal peptide and transmembrane domain, with the exception of Glyma02g03250 (Figure 6; Additional file 1). Several of the putative flower MMP genes contained a GPI-anchor modification site, which was similar to 3 *Arabidopsis* MMP genes, At2-MMP, At4-MMP, At5-MMP [47], and the known soybean MMP gene GmMMP2 (referred to as Gm2-MMP) [42] (Figure 6). None of the putative flower MMP genes or known soybean genes (Gm2-MMP or SMEP1) [48,55,56] contained a furin cleavage site, which was present in several *Arabidopsis* MMP genes. When amino acid sequence similarity identity was compared between all putative flower MMP genes and Gm2-MMP using Clustal W (http://npsa-pbil.ibcp.fr/cgi-bin/npsa_automat.pl?page=/NPSA/npsa_server.html), little homology between the flower MMP and leaf MMP genes was found, with the exception of Glyma01g04350 which showed 99% sequence similarity to Gm2-MMP (data not shown). All putative flower MMP genes, with the exception of Glyma01g04350, had an E (glutamate) to Q (glutamine) residue substitution in the zinc-binding motif of the catalytic domain, which has been identified in other legume species [51]. The glutamate residue is required for functional protease activity [57], thus the amino acid switch in soybean flower MMPs may render these inactive. Still, they may be important for O_3_ stress response because experiments with *Medicago truncatula* have also demonstrated a functional role for proteolytically-inactive MMPs in biotic stress response [51].

The responsiveness of putative soybean flower MMPs to elevated [O_3_] is consistent with the ECM being the primary point of O_3_ contact within plant cells and the location where antioxidant metabolism begins to protect cells from ROS damage [34,58]. Stress-responsive signaling pathways, including jasmonic acid, salicylic acid and ethylene-dependent redox signaling are all triggered by the redox sensing that occurs in the ECM [34]. The soybean MMP gene Gm2-MMP was up-regulated consistently with the release of ROS during pathogenic infection [50], possibly linking ROS signaling and MMP gene expression in soybean stress response. Previous analysis of Arabidopsis MMP gene expression revealed that At3-MMP was expressed at greater abundance in response to O_3_ treatment, with a slight increase in At2-MMP in response to O_3_ as well [59]. While the putative MMP genes are present in high abundance in flower tissues exposed to elevated [O_3_], analysis of the expression profiles of the putative MMP genes in soybean using RNA-Seq Atlas (http://soybase.org/soyseq/) found that these genes were not present, or present in low abundance in other soybean tissues. Therefore, it is hypothesized that the increase in abundance of the putative MMP genes identified in this study may represent a distinct flower response to O_3_ stress in soybean.

**Figure 7:**
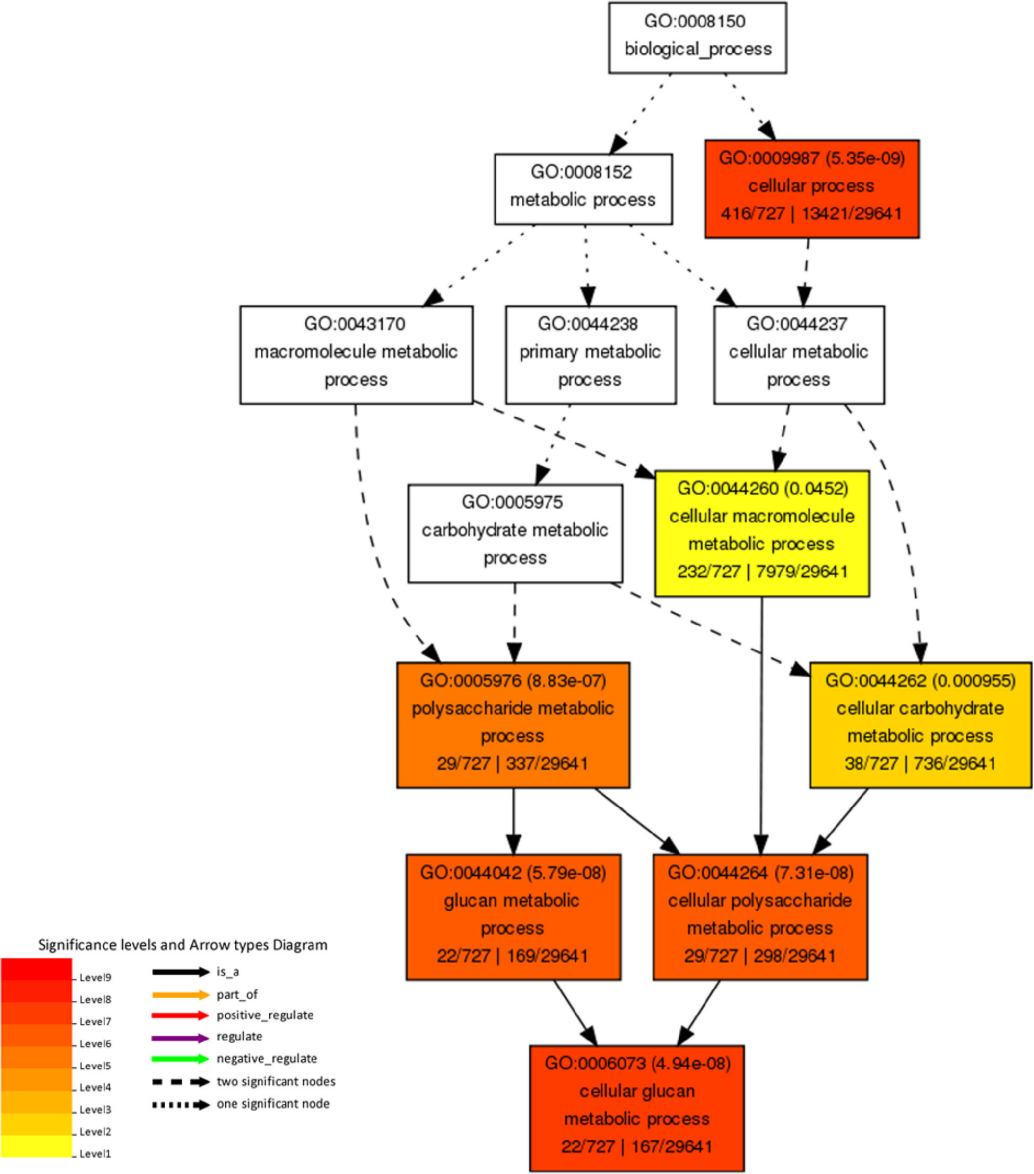
**Analysis of gene ontology (GO) term enrichment of biological processes containing XTH genes in pod tissues.** Biological terms with increasing overrepresentation in pod tissues exposed to elevated [O_3_] are represented by increasingly red colors. GO term enrichment was performed using single enrichment analysis (SEA) tool on AgriGo (http://bioinfo.cau.edu.cn/agriGO/).

In pod tissue, cell wall modification and calcium signaling genes showed the greatest increase in abundance in response to elevated [O_3_] (Table 2). Gene ontology (GO) enrichment analysis of biological processes was performed for genes differentially expressed only in pod tissue. Apoptosis, signal transduction, ATP biosynthetic processes, cellular glucan metabolic processes, protein amino acid phosphorylation and innate immune responses were enriched in pod tissue (Additional file 2). These activities are known to increase in plants in response to both abiotic and biotic stress [60-65], and the possibility that O_3_ stress co-opts pathways involved in biotic stress response has been previously proposed [66,67]. The genes with the greatest increase in abundance in response to elevated [O_3_] were xyloglucan endotransglucosylase/hydrolase family proteins (XTH) (Table 2). Genes annotated as XTHs also had high mean expression, along with the greatest increase in abundance in response to O_3_ in pod tissue (Figure 2). These genes belong to the GO biological process of cellular glucan metabolic processes, which is highly enriched in pod tissues (Figure 7). Analysis of the putative XTH genes in soybean using RNA-Seq Atlas (http://soybase.org/soyseq/) showed that these genes were not present or in low abundance in other tissues, indicating that these genes may represent a distinct pod response to elevated [O_3_].

**Table 2:** **Genes with the greatest log fold change in response to elevated [O**_**3**_ **] in pod tissues**

**Gene**	***p*** **- value**	**Log**_**2**_ **fold change**	**Functional group**	**Description**
Glyma17g07260	0.043	2.91	Cell wall	Xyloglucan endotransglucosylase/hydrolase family protein
Glyma17g07240	0.043	2.91	Cell wall	Xyloglucan endotransglucosylase/hydrolase family protein
Glyma13g01120	0.035	2.83	Cell wall	Xyloglucan endotransglucosylase/hydrolase family protein
Glyma17g07250	0.043	2.55	Cell wall	Xyloglucan endotransglucosylase/hydrolase family protein
Glyma17g07280	0.046	2.52	Cell wall	Xyloglucan endotransglucosylase/hydrolase family protein
Glyma01g03005	0.022	2.45	NA	NA
Glyma13g01131	0.038	2.36	Cell wall	Xyloglucan endotransglucosylase/hydrolase family protein
Glyma13g01140	0.042	2.33	Cell wall	Xyloglucan endotransglucosylase/hydrolase family protein
Glyma17g07220	0.039	2.32	Cell wall	Xyloglucan endotransglucosylase/hydrolase family protein
Glyma06g10700	0.043	2.23	Signaling	Phosphate-responsive 1 family protein
Glyma06g11700	0.038	2.20	RNA	AP2 domain
Glyma13g01110	0.043	2.20	Cell wall	Xyloglucan endotransglucosylase/hydrolase family protein
Glyma17g07270	0.043	2.13	Cell wall	Xyloglucan endotransglucosylase/hydrolase family protein
Glyma12g31150	0.050	2.09	Development	No apical meristem (NAM) protein
Glyma18g03066	0.026	2.06	Signaling	Leucine-rich repeat receptor-like protein kinase
Glyma13g05090	0.026	2.02	NA	NA
Glyma10g37510	0.014	2.02	Transport	Heavy metal associated protein
Glyma13g35950	0.027	2.00	Signaling	Calcium-binding EF hand family protein
Glyma08g04920	0.017	1.99	Signaling	Calcium binding protein-like
Glyma11g35334	0.022	1.96	Protein	Leucine-rich repeat receptor-like protein kinase
Glyma12g01420	0.021	1.95	Stress	NB-ARC domain-containing disease resistance protein
Glyma10g37500	0.021	1.94	NA	Heavy metal associated protein
Glyma14g22970	0.043	1.92	RNA	AP2 domain
Glyma12g34580	0.017	1.91	Signaling	Calcium-binding EF-hand family protein
Glyma02g04620	0.027	1.90	Transport	Mitochondrial carrier protein

‘NA’ indicated genes not assigned an annotation. FDR-adjusted *p*-values are shown.

XTH is a well-known cell wall-modifying enzyme that plays a role in growth and differentiation in plants [68]. Genes in the XTH family are involved in cell elongation in vascular cells [69,70], epidermal cells [71], inflorescence apices [72], primary roots [73] and during somatic embryogenesis [74]. XTH also plays a role in floral organ abscission [75,76]. Due to the similarity of flower abscission and pod dehiscence zone [77] and the known response of XTH genes to oxidative [78], water [79] and biotic stress [80], it is hypothesized that XTH genes may play a role in pod dehiscence in soybean exposed to elevated [O_3_].

## Conclusion

Soybean is an O_3_-sensitive crop, with current tropospheric [O_3_] costing billions of dollars in lost production annually. In this study, it was established that gene expression in reproductive tissues in soybean is altered by elevated [O_3_]. There were 4,703 transcripts responsive to elevated [O_3_] in both flower and pod tissues, yet those genes did not respond consistently in the two tissues. This indicates that reproductive tissues have more distinct than similar transcriptomic responses to elevated [O_3_]. Flower tissues responded to elevated [O_3_] through increased expression of MMP genes. It was notable that these flower MMP genes may not be proteolytically active based on amino acid composition, but they clearly respond to O_3_ stress. Pod tissues responded to elevated [O_3_] through increased expression of cell expansion genes. The increased transcript abundance of XTH genes supports a role of these genes in pod dehiscence in soybean exposed to elevated [O_3_].

## Methods

### Growth chamber experimental design and conditions

Soybean (*Glycine max* L. Merr. cv. 93B15; Pioneer Hi-Breed) was grown in ambient air (<20 ppb) and elevated ozone (150 ppb) in 14 h/10 h day/night schedules under PPFD of ~650-750 μmol m^−2^ s^−1^; RH 60%; 25°C day/21°C night conditions in 8 growth chambers (Conviron, Winnipeg, Manitoba, Canada). Soybean plants were grown in 6-L pots (Classic C600, Nursery Supplies, Chambersburg PA, USA) in sterilized soil (LC-1 Sunshine Mix (SunGro Horticulture Canada Ltd, Bellevue, WA, USA)) and treated with 50% Long Ashton solution supplemented with 3 mM NH_4_NO_3_ [81]. Two seeds were planted per pot ~4 cm below the soil surface and then thinned to one plant per pot once seeds successfully germinated. A total of 12 plants were grown per chamber in a randomized complete block design (n = 4). Plants were rotated among chambers once a week and within chambers every two days to minimize chamber effects.

### Tissue sampling and molecular analyses

Tissue sampling for RNA was done during R2 (full bloom) and R4 (full pod) for growth chamber grown plants. Plants were considered at full bloom when there was an open flower at one of the first two uppermost nodes with a fully expanded leaf. Plants were considered at full pod when there was a pod 2 cm in length present on one of the four uppermost nodes with a fully expanded leaf. At each stage the appropriate tissue was sampled (full open flowers at R2 and initiating pods at R4). Sampling was done at the nodes 2–4 (from the top of the plant) in order to avoid compensation and senescence effects on the upper and lowermost nodes. Tissue from four plants was sampled per developmental stage per block. Immediately after collection flower and pod tissue was plunged into liquid N and stored at −80°C. Flower or pod tissue was ground to a fine power using a mortar and pestle.

Total RNA was extracted from ground tissue using PureLink Plant RNA Reagent (Ambion, by Life Technologies Corp., Grand Island, NY, USA) according to the manufacturer’s protocol. RNA quantity was determined with a spectrophotometer (Nanodrop 1000, Thermo Fischer Scientific, Waltham, MA, USA) and RNA quality was assessed using the Agilent 2100 Bioanalyzer (Agilent Technologies, Santa Clara, CA, USA) on an RNA Nano chip. Genomic DNA contamination was removed from RNA samples using Turbo DNase treatment (Applied Biosystems/Ambion, Austin, TX, USA) according to the manufacturer’s protocol. cDNA library preparation was done using the Illumnina TruSeq Sample Prep kits (Illumina Inc. San Diego, CA, USA). Each library fragment was barcoded during library preparation and multiplexed for sequencing. Tissue samples per block (4 subsamples) were pooled for a total of 8 libraries prepared for each tissue (16 libraries total).

### RNA-sequencing (RNA-seq), bioinformatics and statistical analysis

Sequencing was done at the Roy J. Carver Biotechnology Center using the Illumina Genome HiSeq 2000 (Illumina Inc. San Diego, CA, USA, http://www.illumina.com) and Cassava pipeline 1.8 to obtain 100 nt single-end reads. Samples were sequenced in groups of 4 across 4 lanes and generated ~31-63 million reads per sample. All FASTQ files from all sequencing runs are located in the Small Read Archive (http://www.ncbi.nlm.nih.gov/sra), SRP035871, BioProject number PRJNA236472. Quality control for reads generated from sequencing was performed using FastQC (http://www.bioinformatics.babraham.ac.uk/projects/fastqc/). Sequenced reads were aligned to the soybean reference genome (Gmax_189.fa, www.phytozome.net) using Bowtie [82]. All valid alignments per read were reported allowing up to three mismatches. Alignment summary statistics are presented in Additional file 3. Aligned sequence reads and a list of genomic features (Gmax_189_gene.gff3, www.phytozome.net) were input into HTSeq to generate read counts using the htseq-count and –m union options. These counts were then input into SAS (SAS Institute, Version 9.2, Cary, NC, USA) for normalization and statistical analysis. Genes with counts of 10 or less were removed from all subsequent statistical analyses. Read counts were normalized using the natural log of the upper quartile (ln_uq) [83,84]. All count data can be found in Additional files 4 and 5. Differential gene expression was determined using a mixed effects linear model Y_ijkl_ = m + t_i_ + γ_j_ + ρ_k_ + ɛ_ijkl_. Y is the normalized estimate of the expression for the fixed effect of condition (i = ozone/ambient), the random effect of block (j = 1,2,3,4) and the random effect of lane (k = 1,2,3,4). A log fold change represents the difference of the ln_uq normalized count data for elevated [O_3_] minus ambient [O_3_]. The assumptions of normality were tested using the Shapiro-Wilk test [85] for each gene. A multiple test correction was applied using the linear step-up method of [86]. Analyses were conducted in SAS (SAS Institute, Version 9.2, Cary, NC, USA).

### Availability of supporting data

The data set supporting the results of this article are included within the article (and its additional files). Additionally, all FASTQ files from all sequencing runs are located in the Small Read Archive (http://www.ncbi.nlm.nih.gov/sra), SRP035871, BioProject number PRJNA236472. .

## Additional files

Additional file 1:
**Domain analysis of putative MMP genes found in flower tissue.** The general structure (domain analysis) of all members of the *Arabidopsis* MMP family (At1-MMP to At5-MMP), and the two known soybean MMPs (SMEP1 and GmMMP2) was found in [59]. Domain analysis of the putative MMP genes found in flower tissue in our dataset was also completed to compare with known MMP genes. The protein sequence for each gene in our data set was determined using the Phytozome database (http://www.phytozome.net/) and the amino acid length and the presence of a signal peptide, transmembrane and catalytic domain was analyzed using InterPro (https://www.ebi.ac.uk/interpro/). The signal peptide cleavage site and C-terminal transmembrane domain were also analyzed using the predictive software program SignalP 4.1 (http://www.cbs.dtu.dk/services/SignalP/) [87] and Localizome (http://localodom.kobic.re.kr/LocaloDom/), respectively. The presence of a furin cleavage site was analyzed using the predictive software ProP 1.0 (http://www.cbs.dtu.dk/services/ProP/) and the presence of a GPI anchor domain was analyzed using the predictive software big-PI Plant Predictor (http://mendel.imp.ac.at/gpi/plant_server.html). The cysteine switch and zinc-binding motifs of putative soybean flower MMP genes were determined using sequence alignment with known *Arabidopsis* and soybean MMP genes and generated using PRALINE (http://www.ibi.vu.nl/programs/pralinewww/) [88]. Percent identity of amino acid sequence analysis was performed Network Protein Sequence Analysis (http://npsa-pbil.ibcp.fr/cgi-bin/npsa_automat.pl?page=/NPSA/npsa_server.html) [89]. Modification sites (signal cleavage and GPI-anchor) are predicted to occur between the given locations of the residues in the amino acid sequence shown in the table. The domain of the GPI-anchor modification is also given and predicted to occur at one of the two bolded and underlined residues. The putative soybean MMP gene Glyma02g03301 has two cysteine switch motifs and two zinc-binding motifs, which is indicated in the table. Additional file 2:
**Gene ontology (GO) term enrichment of biological processes in pod tissue only.** GO term enrichment performed using single enrichment analysis (SEA) tool on AgriGo (http://bioinfo.cau.edu.cn/agriGO/). Additional file 3:
**Summary statistics for each FASTQ file aligned to the soybean reference genome.** The alignment statistics generated from Bowtie are presented in this table. Additional file 4:
**Differentially expressed genes in flower tissue, including the fold change in elevated [O**_**3**_ **], the FDR-adjusted** ***p*** **-value and a description of the functional category.**
Additional file 5:
**Differentially expressed genes in pod tissue, including the fold change in elevated [O**_**3**_ **], the FDR-adjusted** ***p*** **-value and a description of the functional category.**

## Abbreviations

O_3_: Ozone
[O_3_]: Ozone concentration
ppb: Part per billion
ROS: Reactive oxygen species
PPFD: Photosynthetic photon flux density
RH: Relative humidity
ECM: Extra-cellular matrix
MMP: Matrix metalloproteinase
XTH: Xyloglucan endotransglucosylase/hydrolase
