# Supplementary material for: Distinct transcriptional profiles of ozone stress in soybean (Glycine max) flowers and pods
Source: BMC Plant Biol. 2014 Nov 28;14:335. doi: 10.1186/s12870-014-0335-y (PMC4263021; doi:10.1186/s12870-014-0335-y)
Supplement: Additional file 1: — Domain analysis of putative MMP genes found in flower tissue. The general structure (domain analysis) of all members of the Arabidopsis MMP family (At1-MMP to At5-MMP), and the two known soybean MMPs (SMEP1 and GmMMP2) was found in [59]. Domain analysis of the putative MMP genes found in flower tissue in our dataset was also completed to compare with known MMP genes. The protein sequence for each gene in our data set was determined using the Phytozome database (http://www.phytozome.net/) and the amino acid length and the presence of a signal peptide, transmembrane and catalytic domain was analyzed using InterPro (https://www.ebi.ac.uk/interpro/). The signal peptide cleavage site and C-terminal transmembrane domain were also analyzed using the predictive software program SignalP 4.1 (http://www.cbs.dtu.dk/services/SignalP/) [87] and Localizome (http://localodom.kobic.re.kr/LocaloDom/), respectively. The presence of a furin cleavage site was analyzed using the predictive software ProP 1.0 (http://www.cbs.dtu.dk/services/ProP/) and the presence of a GPI anchor domain was analyzed using the predictive software big-PI Plant Predictor (http://mendel.imp.ac.at/gpi/plant_server.html). The cysteine switch and zinc-binding motifs of putative soybean flower MMP genes were determined using sequence alignment with known Arabidopsis and soybean MMP genes and generated using PRALINE (http://www.ibi.vu.nl/programs/pralinewww/) [88]. Percent identity of amino acid sequence analysis was performed Network Protein Sequence Analysis (http://npsa-pbil.ibcp.fr/cgi-bin/npsa_automat.pl?page=/NPSA/npsa_server.html) [89]. Modification sites (signal cleavage and GPI-anchor) are predicted to occur between the given locations of the residues in the amino acid sequence shown in the table. The domain of the GPI-anchor modification is also given and predicted to occur at one of the two bolded and underlined residues. The putative soybean MMP gene Glyma02g03301 has two cysteine switch motifs and two [file 12870_2014_335_MOESM1_ESM.pdf]

Gene	Length (AA)	Signal peptide length (InterPro)	Signal peptide cleavage site (SignalP 4.1)	Cysteine switch motif	Furin cleavage site (ProP 1.0)	Catalytic domain (InterPro)	Zinc-binding domain motif	GPI-anchor modification site	GPI-anchor modification domain (big-PI)	Transmembrane domain (Localizome)
Glyma02g03250	386	1 to 24	24,25	IRCGVPD	NO	197 to 379	HQIGHLLGLD	NO	n/a	NO
Glyma02g03301	759	1 to 24	24,25	IRCGVPD (2)	NO	187 to 356; 465 to 713	HQIGHLLGLD (2)	731,732	NSH **DS**	739 to 758
Glyma02g03320	388	1 to 24	24,25	LRCGVPD	NO	147 to 333	HQIGHLLGLD	360,361	NSH **DS**	368 to 386
Glyma02g03230	393	1 to 25	23,24	IRCGVPD	NO	164 to 348	HQIGHLLGLE	365,366	NSH **DS**	375 to 392
Glyma01g04370	340	1 to 23	23,24	PRCAVPD	NO	121-310	HQIGHLLGLD	312,314	QQY A**N**G**N**	321 to 339
Glyma02g03335	168	1 to 28	28,29	NO	NO	63 to 141	NO	NO	n/a	NO
Glyma0420s50	161	NO	NO	NO	NO	63 TO 138	HQIGHLLGLD	NO	n/a	NO
Glyam02g03210	356	1 to 24	24,25	LRCGVPD	NO	71 TO 323	HQIGHLLGLD	328,320	GFA **NS**A	333 to 355
Glyma01g04350	357	1 to 26	26,27	LRCGVPD	NO	136 to 322	HEIGHLLGLD	329,330	NVE **DS**	334 to 356

Additional file 1.
