# Supplementary material for: Distinct transcriptional profiles of ozone stress in soybean (Glycine max) flowers and pods
Source: BMC Plant Biol. 2014 Nov 28;14:335. doi: 10.1186/s12870-014-0335-y (PMC4263021; doi:10.1186/s12870-014-0335-y)
Supplement: Additional file 3: — Summary statistics for each FASTQ file aligned to the soybean reference genome. The alignment statistics generated from Bowtie are presented in this table. [file 12870_2014_335_MOESM3_ESM.pdf]

Treatment	Tissue	Replicate	Total Reads	Reads Aligned (Bowtie)
Control	Flower	1	47074876	32536378
Ozone	Flower	1	43450568	29219245
Control	Flower	2	31732387	19888810
Ozone	Flower	2	37890303	25567314
Control	Flower	3	36922987	24366434
Ozone	Flower	3	55455297	36950628
Control	Flower	4	36650396	24615450
Ozone	Flower	4	50142261	31117217
Control	Pod	1	32179582	20809724
Ozone	Pod	1	50104192	34361241
Control	Pod	2	43110160	28354201
Ozone	Pod	2	39144705	26476814
Control	Pod	3	63350532	43679202
Ozone	Pod	3	29515055	19272642
Control	Pod	4	47773610	32389776
Ozone	Pod	4	49501945	38309568

Additional file 3.
