# Supplementary material for: Distinct transcriptional profiles of ozone stress in soybean (Glycine max) flowers and pods
Source: BMC Plant Biol. 2014 Nov 28;14:335. doi: 10.1186/s12870-014-0335-y (PMC4263021; doi:10.1186/s12870-014-0335-y)
Supplement: Additional file 2: — Gene ontology (GO) term enrichment of biological processes in pod tissue only. GO term enrichment performed using single enrichment analysis (SEA) tool on AgriGo (http://bioinfo.cau.edu.cn/agriGO/). [file 12870_2014_335_MOESM2_ESM.pdf]

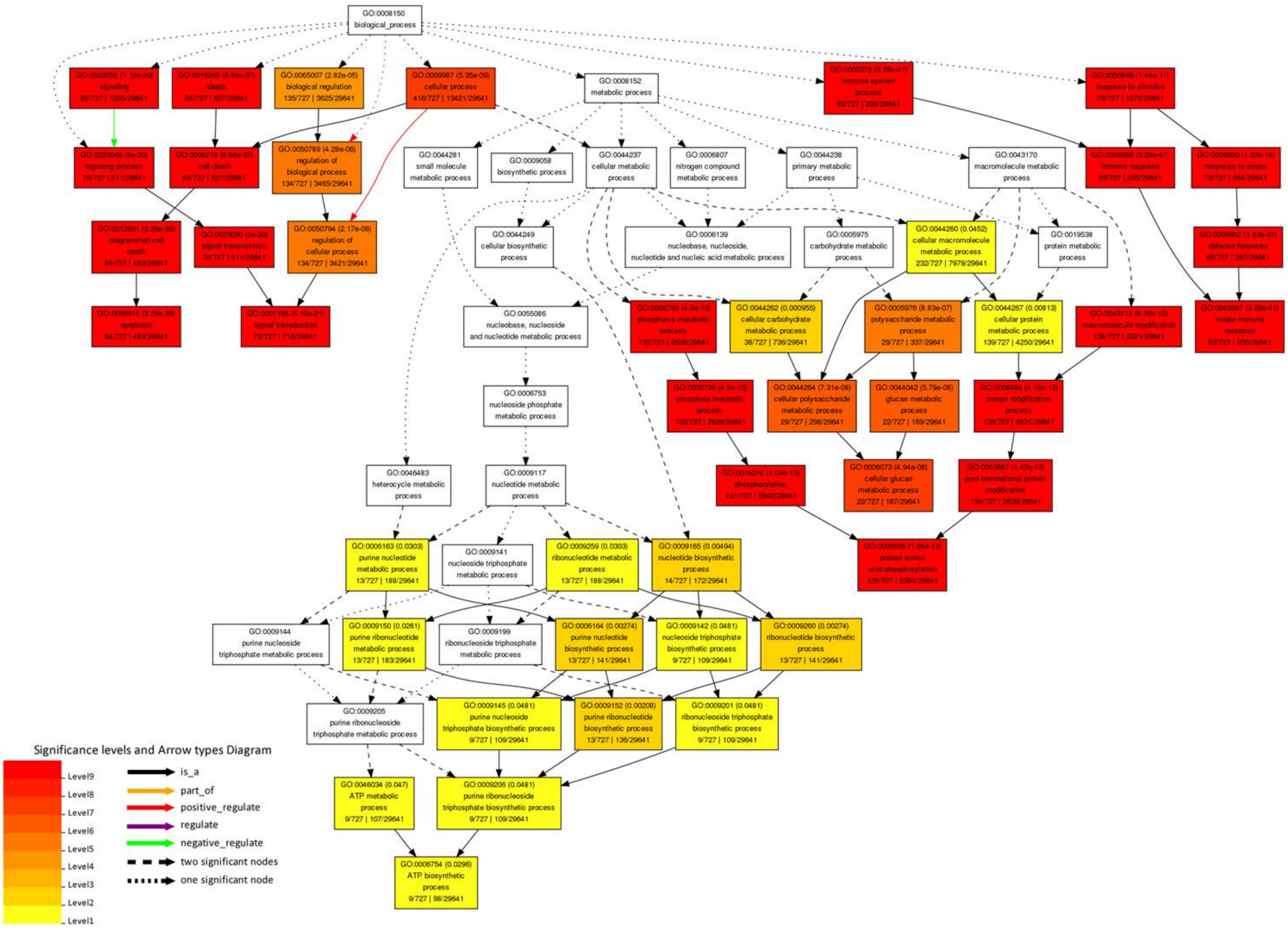

Additional file 2.
